# Clinical correlation of A2063G gene mutation in mycoplasma pneumoniae pneumonia in children: a retrospective analysis

**DOI:** 10.3389/fped.2026.1866647

**Published:** 2026-08-13

**Authors:** Jianshan Huang, Xiating Huang, Zhanpeng Qiu, Hui Li

**Affiliations:** 1 Xiamen TCM Hospital Affiliated to Fujian University of Traditional Chinese Medicine, Fujian, Xiamen, China; 2 Xiamen Hospital, Dongzhimen Hospital, Beijing University of Chinese Medicine, Fujian, Xiamen, China; 3 Fujian University of Traditional Chinese Medicine, Fujian, Fuzhou, China

**Keywords:** A2063G gene, children, Mycoplasma pneumoniae pneumonia, pediatric, TNGS

## Abstract

**Objective:**

To investigate the correlation between the A2063G point mutation in the 23S rRNA resistance gene and the clinical features of Mycoplasma pneumoniae pneumonia (MPP) in children.

**Methods:**

A retrospective analysis was conducted on 279 children diagnosed with MPP and hospitalized at Xiamen Hospital of Traditional Chinese Medicine from 2023. Based on the results of drug resistance gene sequencing, the patients were divided into a non-mutation group and a mutation group, and their clinical data were compared.

**Results:**

No significant differences were observed between the two groups regarding symptoms of productive cough and wheezing, or in laboratory indicators including white blood cell count (WBC), creatine kinase (CK), creatine kinase-MB (CK-MB), alanine transaminase (ALT), aspartate transaminase (AST), blood urea nitrogen (BUN), and serum creatinine (Scr) (*P* > 0.05). The total fever duration and hospital stay were significantly longer in the mutation group than in the non-mutation group (*P* < 0.05). Levels of C-reactive protein (CRP), D-dimer (D-D), and lactate dehydrogenase (LDH) were significantly higher in the mutation group (*P* < 0.05). No significant differences between the two groups of children regarding antibiotic regimen selection and the use of fiberoptic bronchoscopy (*P* > 0.05). The usage rate of glucocorticoids in the mutation group was significantly higher than that in the non-mutation group, and the difference was statistically significant (*P* < 0.05). Multivariate analysis indicated that elevated levels of CRP, D-D, LDH and Glucocorticoid Therapy were independently associated with the A2063G mutation.

**Conclusion:**

The A2063G mutation may be associated with prolonged hospital stay, total fever duration, glucocorticoid use, and elevated serum CRP, D-D, and LDH levels. Furthermore, certain independent correlations are present between these clinical parameters. Early identification of this mutation during the initial stages of the disease could provide crucial guidance for adjusting clinical management strategies.

## 1 Introduction

Mycoplasma pneumoniae (MP) is a globally distributed pathogen that has been causing persistent epidemics in various regions for many years. Among children and adolescents, Mycoplasma pneumoniae pneumonia (MPP) has become one of the most common types of community-acquired pneumonia (CAP) (1). Studies from multiple regions have reported a high prevalence of macrolide resistance in pediatric MPP (2–5), posing significant challenges in clinical practice, including suboptimal antibiotic efficacy and prolonged hospitalization. In recent years, targeted next-generation sequencing (tNGS) of respiratory pathogens has emerged as a significant advancement in the diagnosis of pulmonary infections. This method enables highly sensitive and specific detection and analysis of specific pathogenic microorganisms and their associated resistance genes in clinical samples, demonstrating substantial clinical application value (6). This study retrospectively analyzed 279 children diagnosed with MPP and hospitalized at Xiamen Hospital of Traditional Chinese Medicine, aiming to explore the correlation between the A2063G point mutation in the 23S rRNA resistance gene and clinical features, thereby providing reference for clinicians in diagnosis and treatment.

## 2 Materials and methods

### 2.1 Study population

Clinical data from 279 children diagnosed with MPP and hospitalized in the Department of Pediatrics at Xiamen Hospital of Traditional Chinese Medicine from 2023 were retrospectively analyzed. The cohort included 145 males and 134 females, with a mean age of 6.1 years. Based on the results of respiratory pathogen tNGS, they were categorized into a non-mutation group (*n* = 52) and a mutation group (*n* = 227). This study was approved by the Medical Ethics Committee of Xiamen Hospital of Traditional Chinese Medicine (ethical approval number: 2024-X041-01). Given the retrospective nature of this study and the use of anonymized clinical data, the requirement for written informed consent was waived by the Ethics Committee.

### 2.2 Inclusion and exclusion criteria

Inclusion criteria: (1) Meeting the diagnostic criteria outlined in the Guidelines for the Diagnosis and Treatment of Mycoplasma pneumoniae Pneumonia in Children (2023 Edition) (7); (2) Having undergone respiratory pathogen tNGS testing; (3) Possessing complete clinical data; (4) Strict adherence to medical ethics.

Exclusion criteria: (1) Underlying conditions such as cardiac insufficiency or congenital heart disease; (2) Co-infections with tuberculosis, fungi, bacteria, or other viruses; (3) Hospitalization within the past month due to confirmed MP infection; (4) History of severe pneumonia with incomplete recovery; (5) Significant impairment of liver, kidney, or cardiac function.

### 2.3 tNGS detection and grouping method

The laboratory employed targeted next-generation sequencing (tNGS) based on multiplex PCR, with a detection limit as low as 100 copies/mL. Respiratory specimens were collected into preservative-containing sampling tubes and transported under cold-chain conditions at 4 °C to KingMed Clinical Laboratory. The KM MiniSeqDx-CN sequencing platform was utilized, with specific primers designed against 198 respiratory pathogens and 23S rRNA resistance genes. Library construction was performed through two rounds of PCR. The prepared libraries had a concentration of ≥0.5 ng/μL and a fragment size of 250–350 bp, which were subsequently pooled in equal masses, diluted, and subjected to sequencing. The entire detection process incorporated Q30 quality control (corresponding to a base-calling error rate of 0.1% and an accuracy of 99.9%), internal reference data quality control, negative controls (specimens free of pathogen nucleic acids), and positive controls (specimens containing pathogen nucleic acids) to ensure the accuracy of the results.

Based on respiratory pathogen tNGS results, children carrying the A2063G point mutation in the 23S rRNA gene were assigned to the mutation group, while the others constituted the non-mutation group.

### 2.4 Observation indicators

Demographic data (gender, age), clinical parameters (hospital stay, total fever duration, treatment), mutation status of the A2063G locus, symptoms (productive cough, wheezing), and laboratory indicators including white blood cell count (WBC), C-reactive protein (CRP), D-dimer (D-D), lactate dehydrogenase (LDH), creatine kinase (CK), creatine kinase-MB (CK-MB), alanine transaminase (ALT), aspartate transaminase (AST), blood urea nitrogen (BUN), and serum creatinine (Scr) were collected.

### 2.5 Statistical analysis

Statistical analyses were performed using IBM SPSS Statistics for Windows, Version 24.0. A *P*-value of less than 0.05 was considered statistically significant. Categorical variables were expressed as frequencies (percentages) and compared using the Chi-square (*χ*^2^) test or Fisher's exact test when the expected frequency was less than 5. Continuous variables were expressed as mean ± standard deviation and compared using the independent samples *t*-test. Variables with a *P*-value <0.05 in univariate analysis were considered as candidate predictors for inclusion in the multivariate binary logistic regression model. To avoid multicollinearity among independent variables, the variance inflation factor (VIF) was calculated for each candidate variable, with a VIF < 5.0 indicating no significant multicollinearity. Variables with VIF ≥ 5.0 were excluded and the model was refitted.

## 3 Results

### 3.1 Comparison of baseline characteristics

**Figure 1:**
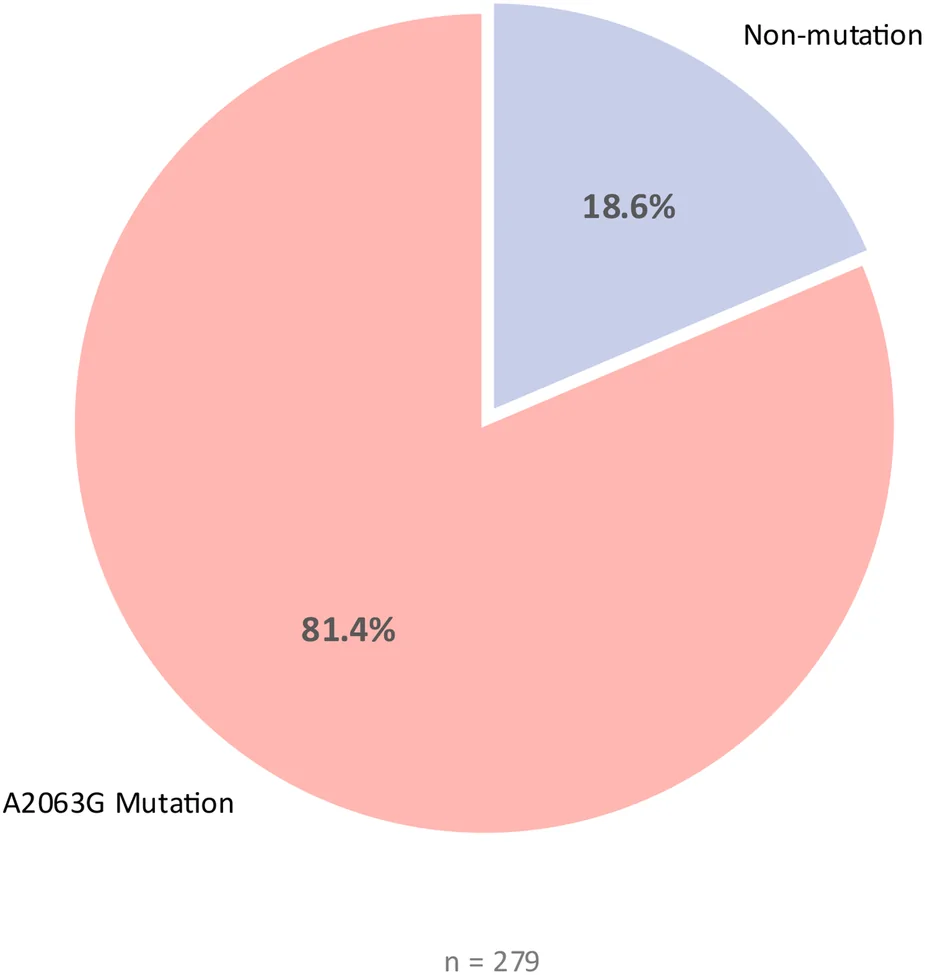
Overall A2063G mutation rate among 279 children with MPP.

Among the 279 children with MPP analyzed, the overall A2063G mutation rate was 81.4%. The non-mutation group consisted of 52 children (26 males, 26 females, mean age 6.1 ± 2.7 years). The mutation group consisted of 227 children (119 males, 108 females, mean age 6.3 ± 2.7 years). No statistically significant differences were found between the two groups regarding gender or age (*P* > 0.05). Details are presented in Figure 1.

### 3.2 Comparison of symptoms and hospital stay duration

**Figure 2:**
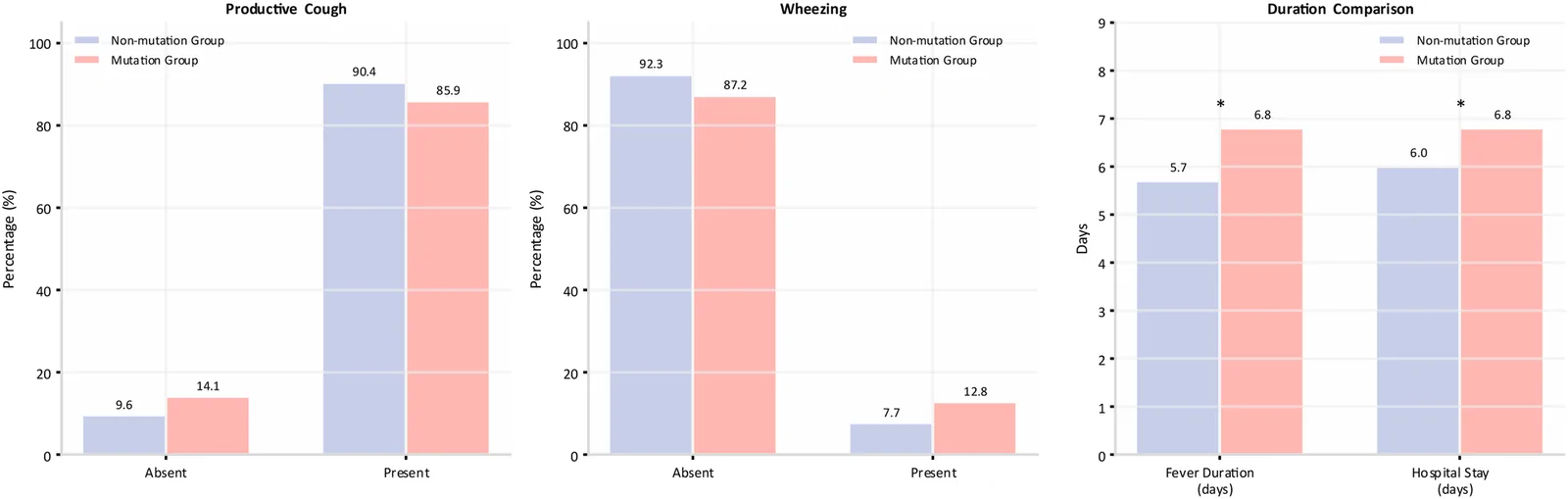
Comparison of symptoms and hospital stay duration between the mutation and mon-mutation groups.

The results showed no significant differences between the two groups in the presence of productive cough or wheezing (*P* > 0.05). The total fever duration and hospital stay were significantly longer in the mutation group compared to the non-mutation group, with the differences being statistically significant (*P* < 0.05). Details are presented in Table 1 and Figure 2.

**Table 1:** Comparison of symptoms and hospital stay duration between the mutation and non-mutation groups (*n*, %).

Indicator	Non-mutation group	Mutation group	*t/χ* ^2^	*P*
Productive cough				
Absent	5 (9.6)	32 (14.1)	0.739	0.390
Present	47 (90.4)	195 (85.9)		
Wheezing				
Absent	48 (92.3)	198 (87.2)	1.048	0.306
Present	4 (7.7)	29 (12.8)		
Total fever duration	5.7 ± 2.6	6.8 ± 3.3	2.171	0.031
Hospital stay	6.0 ± 1.3	6.8 ± 2.6	3.175	0.002

### 3.3 Comparison of treatment

**Figure 3:**
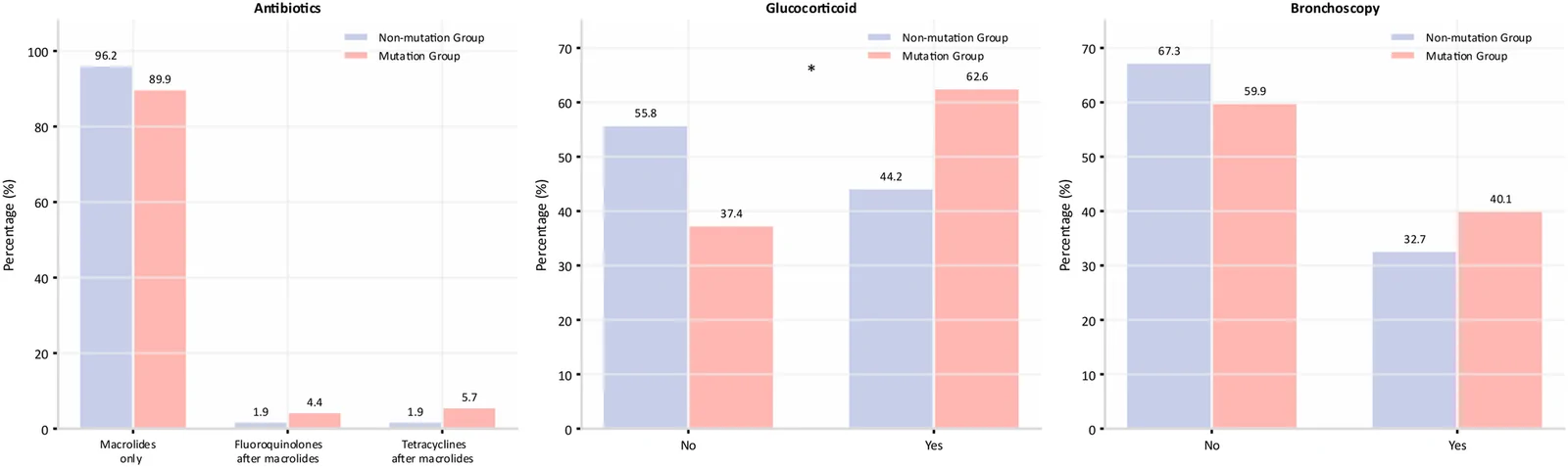
Comparison of treatment between the mutation and non-mutation groups.

The results showed that there were no significant differences between the two groups of children regarding antibiotic regimen selection and the use of fiberoptic bronchoscopy (*P* > 0.05). The usage rate of glucocorticoids in the mutation group was significantly higher than that in the non-mutation group, and the difference was statistically significant (*P* < 0.05). Details are presented in Table 2 and Figure 3.

**Table 2:** Comparison of treatment between the mutation and non-mutation groups (*n*, %).

Indicator	Non-mutation group	Mutation group	*χ* ^2^	*P*
Antibiotics				
Macrolides only	50 (96.2)	204 (89.9)	2.065	0.356
Fluoroquinolones after macrolides	1 (1.9)	10 (4.4)		
Tetracyclines after macrolides	1 (1.9)	13 (5.7)		
Glucocorticoid				
No	29 (55.8)	85 (37.4)	5.879	0.019
Yes	23 (44.2)	142 (62.6)		
Bronchoscopy				
No	35 (67.3)	136 (59.9)	0.975	0.323
Yes	17 (32.7)	91 (40.1)		

### 3.4 Comparison of laboratory indicators

**Figure 4:**
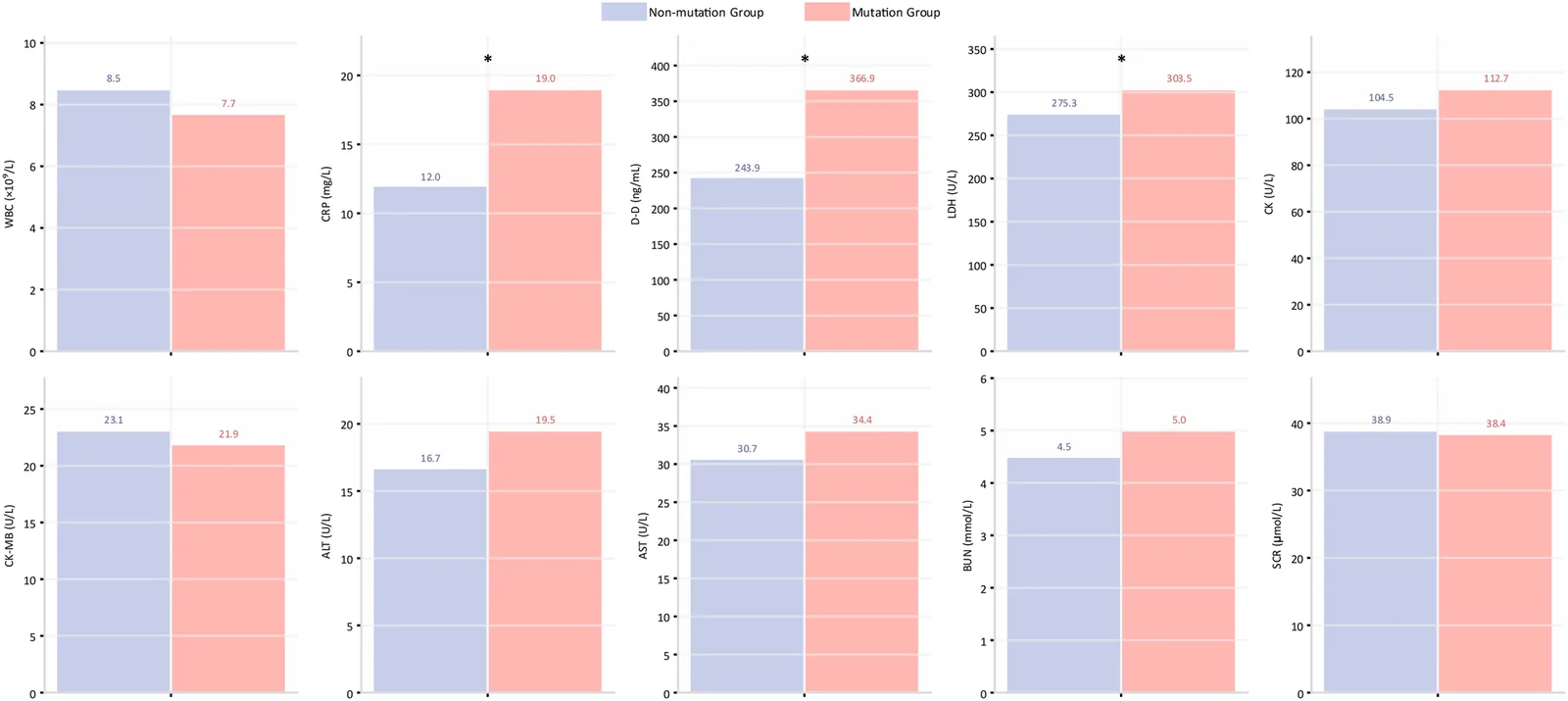
Comparison of laboratory indicators between the mutation and non-mutation groups.

No significant differences were observed between the two groups in WBC, CK, CK-MB, ALT, AST, BUN, or Scr levels (*P* > 0.05). Levels of CRP, D-D, and LDH were significantly higher in the mutation group compared to the non-mutation group, and these differences were statistically significant (*P* < 0.05). Details are presented in Table 3 and Figure 4.

**Table 3:** Comparison of laboratory indicators between the mutation and non-mutation groups.

Laboratory index	Non-mutation group	Mutation group	*t*	*P*
WBC (×10^9^/L)	8.5 ± 4.2	7.7 ± 2.6	1.336	0.186
CRP (mg/L)	12.0 ± 8.8	19.0 ± 16.7	4.257	<0.001
D-D (ng/mL)	243.9 ± 102.6	366.9 ± 254.6	5.570	<0.001
LDH (U/L)	275.3 ± 47.3	303.5 ± 61.9	3.086	0.002
CK (U/L)	104.5 ± 60.3	112.7 ± 89.9	0.624	0.533
CK-MB (U/L)	23.1 ± 12.6	21.9 ± 6.7	0.945	0.346
ALT (U/L)	16.7 ± 7.2	19.5 ± 33.6	0.607	0.545
AST (U/L)	30.7 ± 6.8	34.4 ± 18.3	1.428	0.155
BUN (mmol/L)	4.5 ± 6.1	5.0 ± 8.1	0.373	0.710
SCR (μmol/L)	38.9 ± 12.8	38.4 ± 11.4	0.315	0.753

### 3.5 Logistic regression analysis

Variables with statistical significance in the univariate analysis were entered into a binary logistic regression model to identify those independently associated with the A2063G mutation. This approach was adopted to effectively control model complexity, avoid overfitting, and improve the stability and interpretability of effect estimates. Prior to model entry, multicollinearity among independent variables was assessed using the variance inflation factor (VIF). All VIF values were less than 1.2, indicating no significant multicollinearity. The results showed that elevated levels of CRP, D-D, LDH and Glucocorticoid Therapy were independently associated with the A2063G mutation. Details are presented in Table 4.

**Table 4:** Binary logistic regression analysis for the A2063G mutation in the 23S rRNA gene.

Variable	*β*	SE	Wald *χ*^2^	*P*	OR	95% CI
Hospital stay	0.078	0.091	0.747	0.387	1.081	0.905–1.292
Fever duration	0.109	0.062	3.049	0.081	1.115	0.987–1.260
CRP	0.041	0.017	5.695	0.017	1.041	1.007–1.077
D-D	0.005	0.001	11.956	<0.001	1.005	1.002–1.007
LDH	0.007	0.003	4.164	0.041	1.007	1.000–1.014
Glucocorticoid therapy	0.801	0.346	5.360	0.021	2.227	1.131–4.386

## 4 Discussion

As a common pediatric CAP, MPP primarily manifests with fever and cough. Most patients also experience headaches, rhinorrhea, and malaise. The disease typically begins with a non-productive dry cough, which may progress to paroxysmal severe coughing or even wheezing, adversely affecting the child's physical and mental well-being. Therefore, early and effective intervention is crucial (8). In Western medicine, the primary treatment for MPP is anti-infective therapy. Depending on disease severity, adjunctive treatments such as glucocorticoids, intravenous immunoglobulin, or fiberoptic bronchoscopy may be considered. In recent years, the issue of macrolide antibiotic resistance, the first-line therapy for MPP, has become increasingly prominent. Epidemiological data indicate that the global macrolide resistance rate in M. pneumoniae surged from 18.2% to 76.5% between 2000 and 2019, posing a significant challenge to the standardized treatment of MPP (9). Macrolides exert their antibacterial effect by specifically binding to domain V and II of the 23S rRNA, promoting the dissociation of peptidyl-tRNA from the ribosome and thereby inhibiting protein synthesis. Studies have shown that mutations at key sites (e.g., position 2063) in the MP 23S rRNA reduce the affinity of macrolides for their target, significantly diminishing antibacterial efficacy. This constitutes a key molecular basis for macrolide-resistant MPP in children (10). Through a retrospective analysis of 279 children with MPP hospitalized at Xiamen Hospital of Traditional Chinese Medicine, this study systematically investigated Clinical Correlation of A2063G Gene Mutation in Mycoplasma pneumoniae Pneumonia in Children. The mutation detection rate in this study was 81.4%, consistent with the high resistance rates reported in recent studies (2–5). An Italian study in the southern region showed that, between January 2023 and May 2025, macrolide resistance was detected in 7.5% of MP-positive samples, predominantly attributable to the A2063G mutation (96%), with the highest rate observed in patients aged 10–14 years (12.6%) (11). From January 2014 to March 2024 in South Korea, the PCR positivity rates for MRMP with A2063G and A2064G mutations were 76.6% and 0.7%, respectively. The A2063G rate increased significantly from 2019/20 to 2023/24, and the positivity rate decreased significantly with increasing age (12). A multicenter study on the 2023–2024 Mycoplasma pneumoniae epidemic in China showed that the P1-1 genotype was predominant, accounting for 87.3%–94.9% of isolates, with P1-2c and a rare P1-2g variant also detected. Macrolide resistance was identified in 97.1% of strains, predominantly driven by the A2063G mutation in the 23S rRNA gene (13). A global meta-analysis of molecular epidemiology showed that the macrolide resistance rates were highest among the P1-1, 4572, and ST3 genotypes, and these three genotypes were more prevalent in children than in adults, whereas genotypes P1-2, 3562, 3662, and ST14 were more common in adults (14).

We found that children in the mutation group had significantly longer fever duration and hospital stays compared to the non-mutation group, aligning with findings reported by Zhang et al. (15). The underlying mechanism may involve structural alterations at the macrolide binding site in mutant MP, limiting antibiotic efficacy, reducing pathogen clearance, and prolonging the disease course. This not only increases medical costs (16) and the risk of nosocomial infections but also contributes to the strain on public healthcare resources during MPP outbreaks. Furthermore, this study found that CRP, D-D, and LDH levels were significantly higher in the mutation group, and elevated levels of these markers were independently associated with the A2063G mutation. This may be related to delayed pathogen clearance caused by reduced macrolide susceptibility. CRP, an acute-phase protein that increases significantly during infection or tissue injury, plays a key biological role in clearing pathogens and damaged cells by activating the complement system and enhancing phagocyte function. It is widely used to assess the level of inflammation in infectious diseases (17). D-D not only effectively reflects the body's coagulation function and thrombotic tendency but also serves as a clinical indicator for evaluating inflammatory responses (18). LDH is a ubiquitous metabolic enzyme with essential functions in various physiological processes. Normally maintained at relatively low concentrations, LDH is released in large quantities into body fluids under pathological conditions such as tissue inflammation or cellular damage, leading to significantly elevated levels (19). Research indicates that children with severe MPP have higher levels of LDH, D-D, and CRP compared to those with mild MPP. Elevated levels of these three indicators may signal an increased risk of adverse outcomes, and they demonstrate a synergistic effect in prognostic assessment (20). Therefore, these markers warrant particular attention in clinical practice.

In terms of treatment, this study found that the usage rate of glucocorticoids in the mutation group was significantly higher than that in the non-mutation group, suggesting that MPP children carrying the A2063G mutation may require more aggressive anti-inflammatory therapy. This finding is consistent with the significantly elevated inflammatory markers (CRP, D-D, LDH) observed in the mutation group, indicating that the resistance mutation may exacerbate the host inflammatory response, thereby prompting more frequent clinical use of glucocorticoid intervention. It is noteworthy that despite the significantly higher usage rate of glucocorticoids in the mutation group compared to the non-mutation group, the fever duration and hospital stay were still longer in the mutation group. This may be related to the relatively delayed timing of glucocorticoid administration, which was often initiated only after conventional antibiotics proved ineffective and inflammatory markers continued to rise, as well as the fact that glucocorticoids can only partially suppress the inflammatory response but cannot replace effective pathogen clearance. No significant differences were observed between the two groups regarding antibiotic regimen selection or the utilization rate of fiberoptic bronchoscopy. Regarding antibiotics, the proportion of patients switching to fluoroquinolones or tetracyclines was low in both groups, indicating that clinicians did not significantly alter antibiotic strategies when facing suspected resistant MPP. This reflects a persistent reliance on empirical prescribing and delayed treatment adjustment due to delayed reporting of test results in clinical practice. However, this may also be attributed to the local clinical practice where macrolides remain the empirical first-line treatment, as well as age restrictions and safety concerns regarding the use of fluoroquinolones and tetracyclines in children. Furthermore, the similar application rates of bronchoscopy between the two groups suggest that the decision to perform this procedure in MPP children is primarily based on pulmonary imaging findings and respiratory functional status, rather than solely on resistance mutation detection results.

It should be noted that this retrospective analysis was unable to evaluate several important treatment-related variables, including the time interval from symptom onset to antibiotic initiation, the total duration of antibiotic therapy, the dose and timing of glucocorticoid administration, and the specific clinical indications for fiberoptic bronchoscopy. These factors may simultaneously influence the observed disease severity and clinical outcomes. For example, delayed initiation of antibiotics or glucocorticoids may be associated with prolonged fever duration and extended length of hospital stay, and this association is independent of resistance mutation status. Similarly, the decision to perform bronchoscopy may be driven more by the severity of imaging findings than by molecular drug resistance test results. Therefore, the possibility of confounding by these unmeasured treatment variables cannot be excluded, and the observed association between the A2063G mutation and clinical outcomes should be interpreted with caution.

Additionally, the lack of significant differences in respiratory symptoms like productive cough and wheezing between groups suggests that traditional symptom-based indicators have limited predictive value for resistance, underscoring the need for comprehensive evaluation incorporating laboratory tests.

## 5 Limitations

Although this study explored some important procedural issues, it is subject to the following limitations.

First, as a single-center retrospective study, we were unable to fully exclude the influence of residual confounding. Although common clinical and laboratory indicators were included in the analysis, several key variables that may affect disease severity and treatment decisions were not recorded, such as the timing and duration of antibiotic initiation, glucocorticoid dosage and duration, indications for bronchoscopy, radiological severity scores, and out-of-hospital medication history prior to presentation. These unmeasured factors may be simultaneously associated with both the A2063G mutation status and clinical outcomes, thereby influencing the observed associations. For example, children with more severe illness may present with higher inflammatory markers, be more likely to test positive for the resistant mutation, and be more likely to receive glucocorticoid therapy or bronchoscopy; in the absence of prospective standardized data collection, such confounding cannot be completely adjusted for using statistical methods. Therefore, the findings of this study should be regarded as associative rather than causal.

Second, there was a marked imbalance in sample size between the mutation group (*n* = 227) and the non-mutation group (*n* = 52). Although this imbalance reflects the natural distribution of real-world testing data, it has a substantial impact on both the stability of statistical analyses and the generalizability of the findings. From a statistical perspective, the relatively small sample size of the non-mutation group may not only lead to insufficient statistical power, resulting in failure to detect some true between-group differences, but also reduce the precision of effect estimates. In binary logistic regression analysis, severe between-group imbalance may also compromise the stability of model estimation; the limited number of events in the smaller group may affect the standard error estimation of regression coefficients, thereby influencing the reliability of judgments regarding independent associations. From a generalizability perspective, a non-mutation group of only 52 cases may not adequately represent the overall characteristics of this subset of MPP children, and the resulting bias may limit the applicability of the conclusions across different clinical settings and populations. Therefore, caution should be exercised when extrapolating the findings of this study to broader populations.

Third, this study did not employ systematic severity grading of radiological findings and lacked long-term follow-up data, precluding assessment of the impact of this mutation on the long-term prognosis of MPP (such as pulmonary function recovery, resolution of atelectasis, and recurrent respiratory infections). Therefore, future multicenter, large-sample, prospective studies are urgently needed. Standardized data collection should be used to record treatment timing and protocols in detail, and methods such as propensity score matching should be adopted to control for confounding and ensure more balanced sample sizes between groups, so as to more comprehensively and robustly clarify the true clinical significance of the A2063G mutation in pediatric MPP.

## 6 Conclusion

In conclusion, The A2063G mutation may be associated with prolonged hospital stay, total fever duration, glucocorticoid use, and elevated serum CRP, D-D, and LDH levels. Furthermore, certain independent correlations are present between these clinical parameters. Clinicians' attention to changes in these indicators in clinical practice may facilitate early identification of the risk of resistant mutations. Early detection of this mutation during the initial stages of the disease, combined with dynamic assessment of disease severity using inflammatory markers, may contribute to timely adjustment of clinical management strategies, thereby optimizing decisions regarding anti-infective regimens and anti-inflammatory therapy. Meanwhile, clinicians should maintain heightened vigilance regarding disease progression in MPP patients identified with the A2063G mutation, particularly when patients present with markedly elevated CRP, D-D, and LDH or persistent fever, and should actively evaluate the possibility of macrolide resistance and intervene promptly.

## Data Availability

The datasets presented in this article are not readily available due to the sensitive nature of the data (e.g., patient information), access is restricted. Data are available from the corresponding author upon reasonable request and with permission from the Ethics Committee of Xiamen Hospital of Traditional Chinese Medicine. Requests to access the datasets should be directed to 819977146@qq.com.
